# ﻿*Ranunculusmaoxianensis* (Ranunculaceae), a new species from northwestern Sichuan, China, with an emended description of *R.chongzhouensis*, the putative closest ally of the new species

**DOI:** 10.3897/phytokeys.219.96510

**Published:** 2023-02-01

**Authors:** Wen-Qun Fei, Qiong Yuan, Qin-Er Yang

**Affiliations:** 1 Key Laboratory of Plant Resources Conservation and Sustainable Utilization, South China Botanical Garden, Chinese Academy of Sciences, Guangzhou 510655, Guangdong, China South China Botanical Garden, Chinese Academy of Sciences Guangzhou China; 2 University of Chinese Academy of Sciences, Beijing 100049, China University of Chinese Academy of Sciences Beijing China; 3 Center of Conservation Biology, Core Botanical Gardens, South China Botanical Garden, Chinese Academy of Sciences, Guangzhou 510655, Guangdong, China Center of Conservation Biology, Core Botanical Gardens, South China Botanical Garden, Chinese Academy of Sciences Guangzhou China

**Keywords:** Asia, buttercups, chromosome number, *Ranunculus* sect. *Ranunculus*

## Abstract

*Ranunculusmaoxianensis* (Ranunculaceae), a new species from Jiuding Shan in Maoxian county, northwestern Sichuan province, China, is here illustrated and described. The species is morphologically similar to *R.chongzhouensis*, a species also occurring in Sichuan, in having reniform leaves and puberulous receptacles, carpels and achenes, but differs by having leaves adaxially puberulous with shorter appressed hairs 0.16‒0.28 mm long (vs. longer appressed hairs 0.55‒0.85 mm long), larger flowers (1.8‒2 cm vs. 1.4‒1.6 cm in diameter), larger (8‒10 × 5.5‒6.5 mm vs. 6‒7 × 4.5‒5 mm) and widely obovate petals (vs. obovate), more numerous stamens (35‒55 vs. 12‒18), and subglobose gynoecium and aggregate fruit (vs. ellipsoid). The two species are also different in chromosome number and chromosome morphology. *Ranunculuschongzhouensis* has a karyotype of 2*n* = 2*x* = 16 = 10m + 6sm while *R.maoxianensis* has a karyotype of 2*n* = 4*x* = 32 = 16m + 16sm. An emended description of *R.chongzhouensis* is provided, and its geographical distribution is largely extended.

## ﻿Introduction

*Ranunculus* L., comprising approximately 600 species, is the largest genus in the Ranunculaceae and is widely distributed in all continents (Tamura 1995; Hörandl et al. 2005; Paun et al. 2005; Hörandl and Emadzade 2012). In China, one of the centers of species diversity in *Ranunculus*, more than 150 species and 30 varieties are currently recognized in the genus (Wang 1995a, b, 1996, 2007, 2008, 2013, 2015, 2016, 2018, 2019a, b, 2022; Yang 2000; Wang and Gilbert 2001; Wang and Liao 2009; Luo and Zhao 2013; Wang and Chen 2015; Wang et al. 2016; Yuan and Yang 2017a, b, c; Zhang et al. 2020; Fei et al. 2022, 2023a, b). Many taxa in the genus occur in the Hengduan Mountains region in southwestern China, including mainly southeastern Gansu, eastern Qinghai, western Sichuan, southeastern Xizang (Tibet) and northwestern Yunnan, one of the most important biodiversity hotspots worldwide (Zhang et al. 2009; Sun et al. 2017). In total, 56 species and nine varieties of *Ranunculus* are known from that region (Wang 1993, 1995a, b, 2008, 2013, 2015, 2022; Yang 2000; Wang and Gilbert 2001; Wang and Liao 2009; Wang and Chen 2015; Yuan and Yang 2017c).

**Figure 1.**
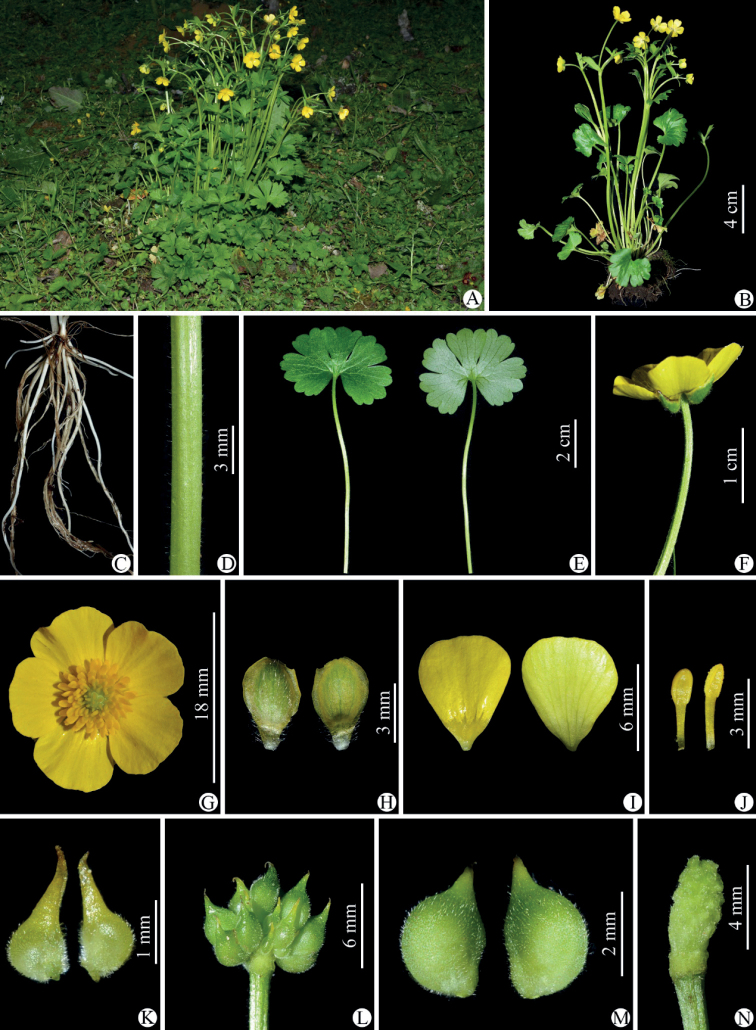
*Ranunculusmaoxianensis* sp. nov. in the wild (China, Sichuan, Maoxian) **A** habitat **B** habit **C** roots **D** portion of stem **E** leaf blade (left: adaxial side; right: abaxial side) **F** flower (lateral view) **G** flower (top view) **H** sepal (left: abaxial side; right: adaxial side) **I** petal (left: adaxial side; right: abaxial side) **J** stamens **K** carpels **L** aggregate fruit **M** achenes **N** receptacle. Photographed by Wen-Qun Fei.

**Figure 2.**
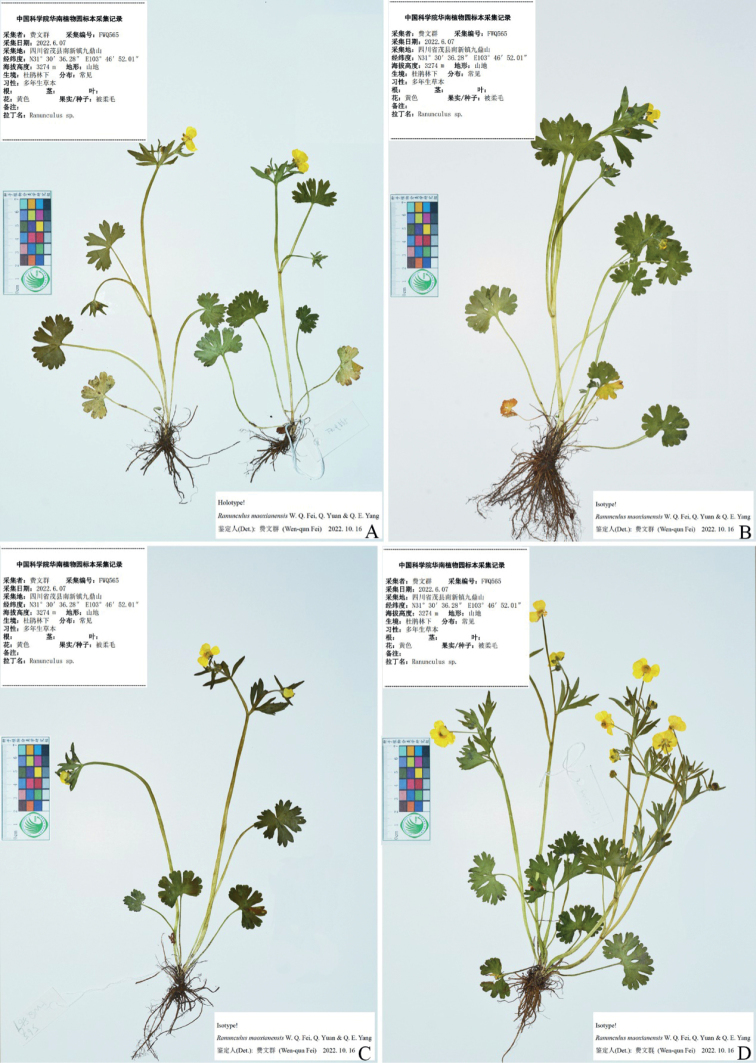
Holotype (**A**) and isotype (**B–D**) sheets of *Ranunculusmaoxianensis* sp. nov.

**Figure 3.**
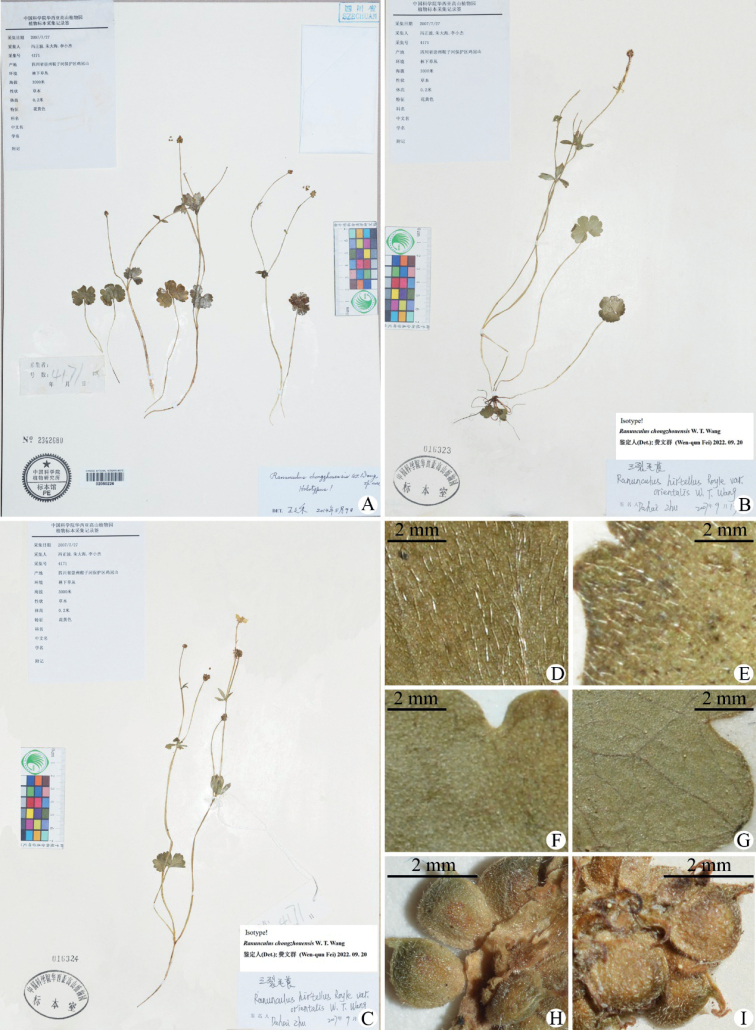
Holotype (**A**) and isotype (**B, C**) sheets of *Ranunculuschongzhouensis*, showing the general morphology and indumentum of leaf blade (**D–G**), aggregate fruit (**H, I**) and receptacle (**H**) **D, E** portion of adaxial side of leaf blade (appressed puberulous with longer hairs) from **A** and **B** respectively **E, G** portion of abaxial side of leaf blade (glabrous) from **A** and **B** respectively **H, I** portion of aggregate fruit (puberulous) from **A** and **C** respectively **H** portion of the receptacle (puberulous) from **A**.

**Figure 4.**
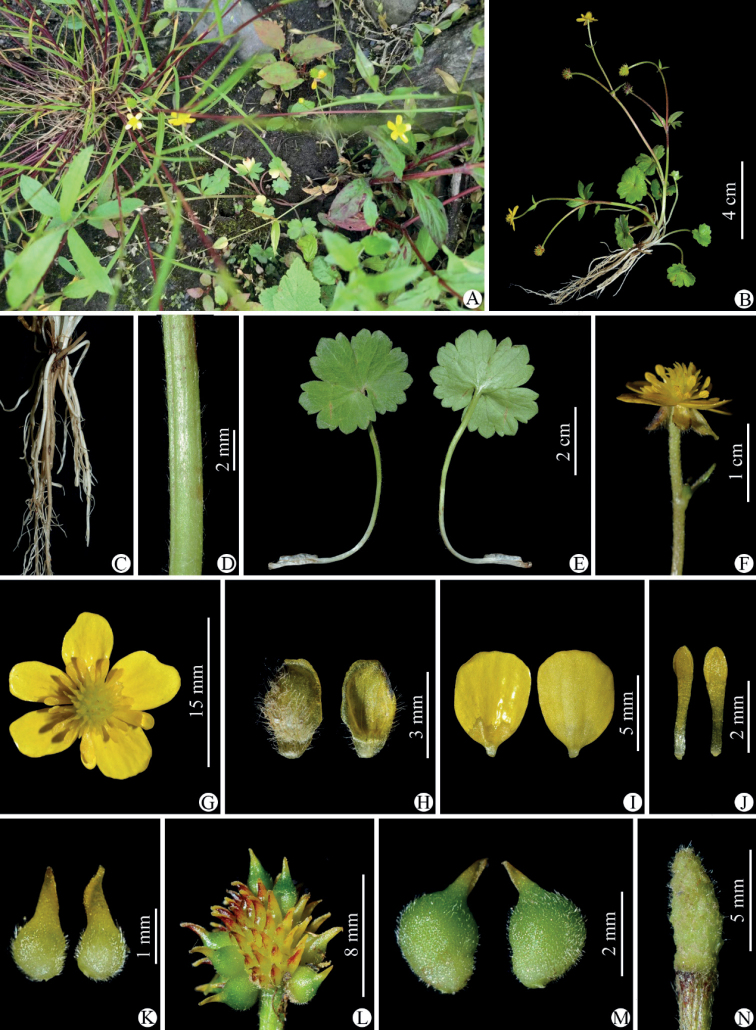
*Ranunculuschongzhouensis* in the wild (China, Sichuan, Chongzhou, the type locality) **A** habitat **B** habit **C** roots **D** portion of stem **E** leaf blade (left: adaxial side; right: abaxial side) **F** flower (lateral view) **G** flower (top view) **H** sepal (left: abaxial side; right: adaxial side) **I** petal (left: adaxial side; right: abaxial side) **J** stamens **K** carpels **L** aggregate fruit **M** achenes **N** receptacle. Photographed by Wen-Qun Fei.

**Figure 5.**
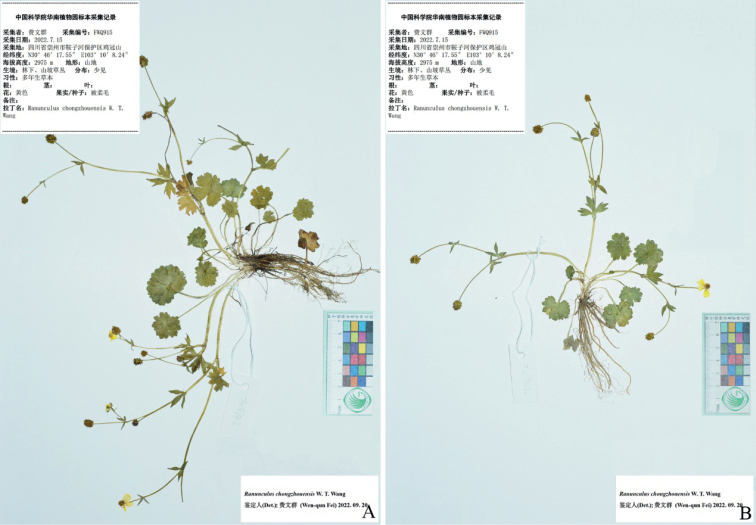
Selected specimens of *Ranunculuschongzhouensis* from its type locality, i.e., Chongzhou city in Sichuan province, China **A, B***W.Q. Fei 915* (IBSC).

**Figure 6.**
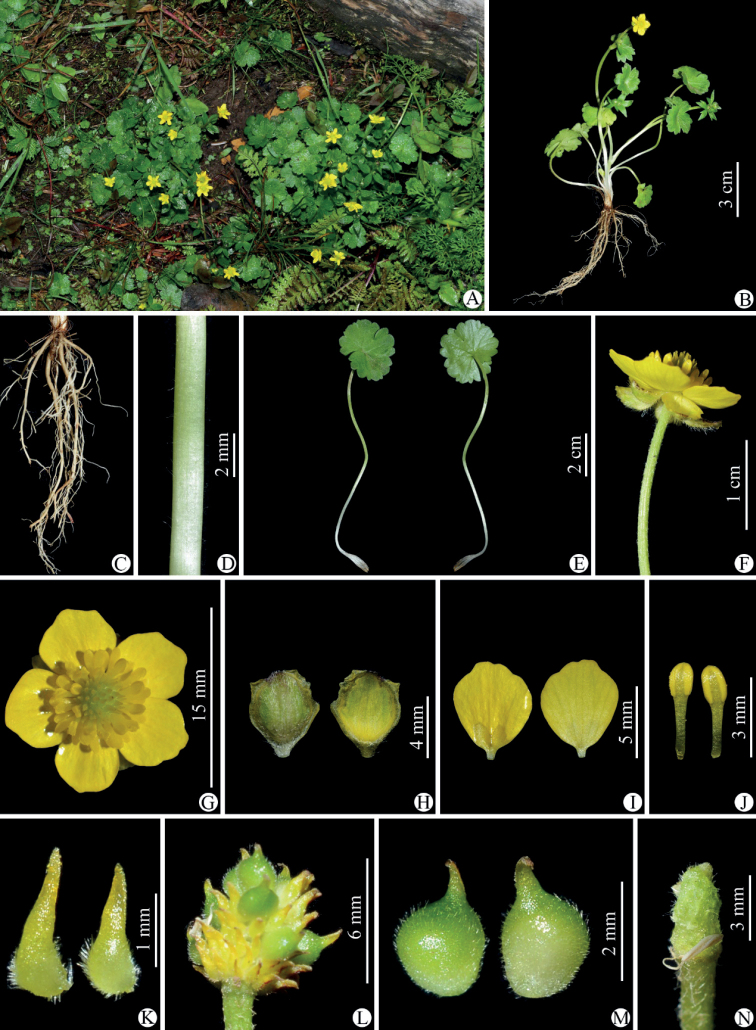
*Ranunculuschongzhouensis* in the wild (China, Sichuan, Dayi) **A** habitat **B** habit **C** roots **D** portion of stem **E** leaf blade (left: adaxial side; right: abaxial side) **F** flower (lateral view) **G** flower (top view) **H** sepal (left: abaxial side; right: adaxial side) **I** petal (left: adaxial side; right: abaxial side) **J** stamens **K** carpels **L** aggregate fruit **M** achenes **N** receptacle. Photographed by Wen-Qun Fei.

**Figure 7.**
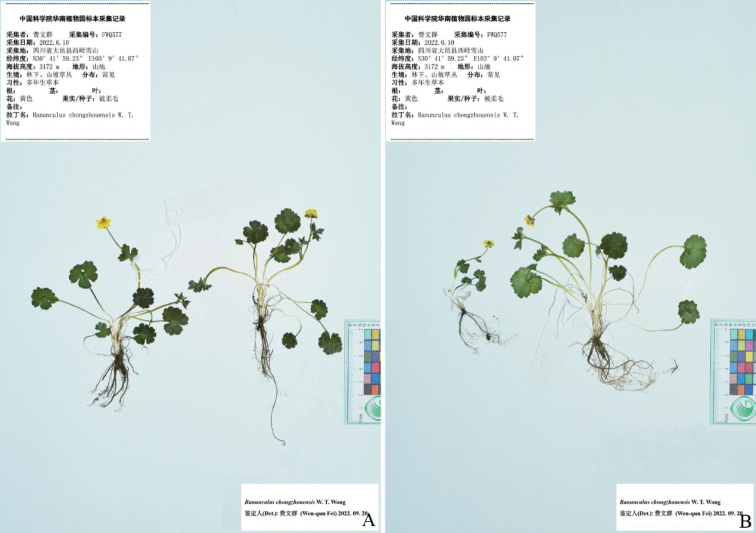
Selected specimens of *Ranunculuschongzhouensis* from Dayi county in Sichuan province, China **A, B***W.Q. Fei 577* (IBSC).

**Figure 8.**
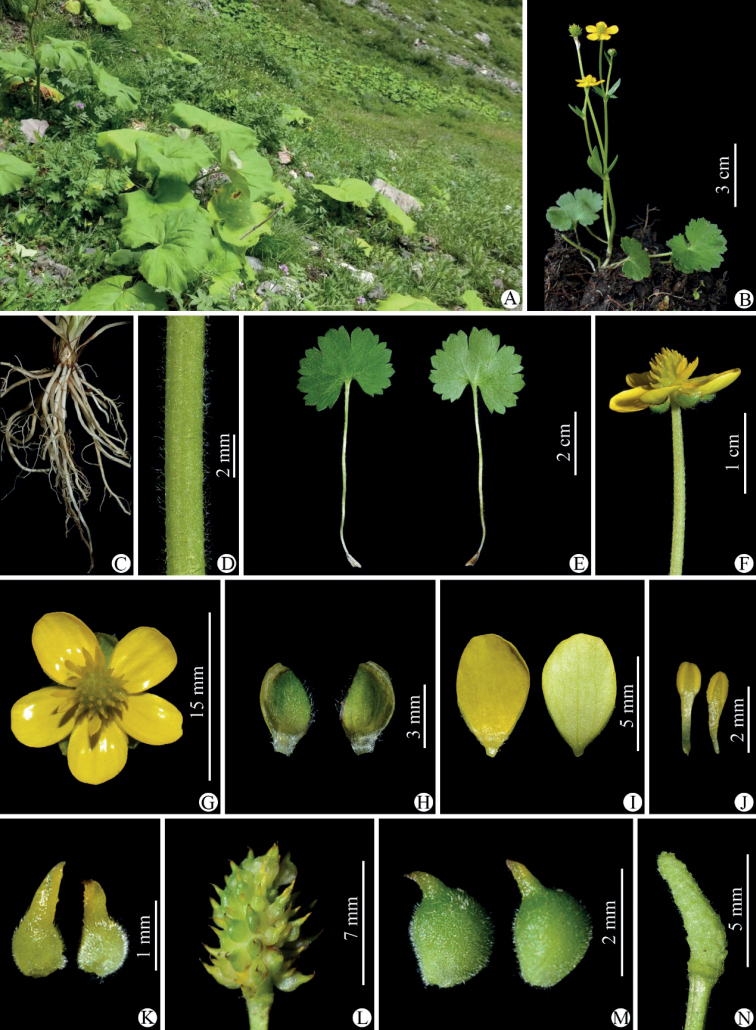
*Ranunculuschongzhouensis* in the wild (China, Sichuan, Xiaojin) **A** habitat **B** habit **C** roots **D** portion of stem **E** leaf blade (left: adaxial side; right: abaxial side) **F** flower (lateral view) **G** flower (top view) **H** sepal (left: abaxial side; right: adaxial side) **I** petal (left: adaxial side; right: abaxial side) **J** stamens **K** carpels **L** aggregate fruit **M** achenes **N** receptacle. Photographed by Wen-Qun Fei.

**Figure 9.**
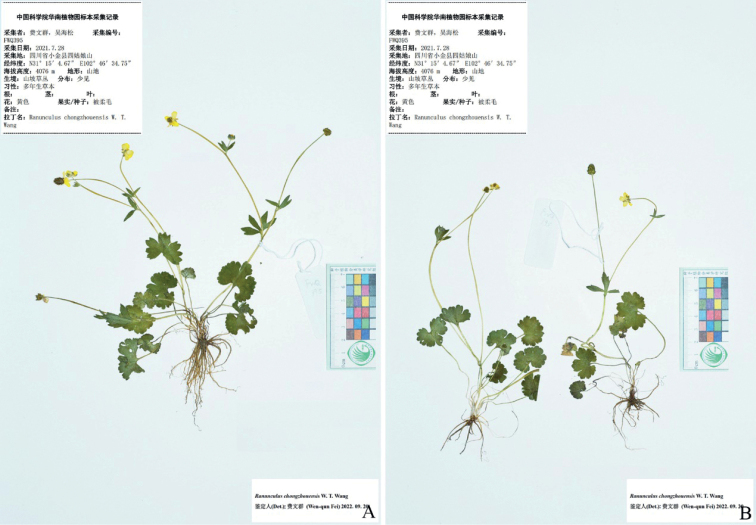
Selected specimens of *Ranunculuschongzhouensis* from Xiaojin county in Sichuan province, China **A, B***W.Q. Fei 395* (IBSC).

**Figure 10.**
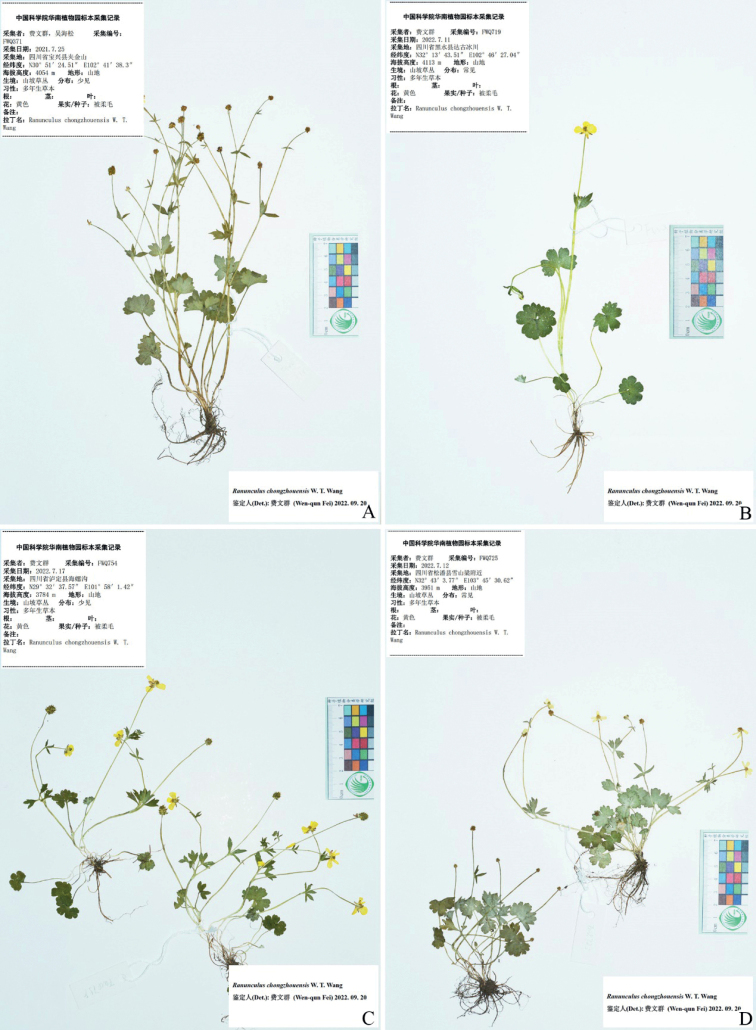
Selected specimens of *Ranunculuschongzhouensis* from Baoxing (**A**), Heishui (**B**), Luding (**C**) and Songpan (**D**) in Sichuan province, China **A***W.Q. Fei 371* (IBSC) **B***W.Q. Fei 719* (IBSC) **C***W.Q. Fei 754* (IBSC) **D***W.Q. Fei 725* (IBSC).

**Figure 11.**
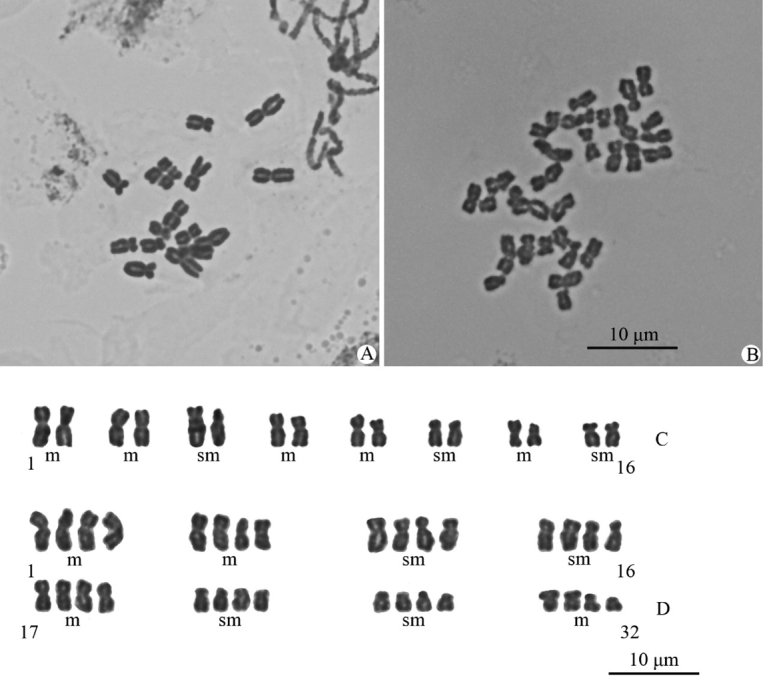
Mitotic metaphase chromosomes (**A, B**) and karyotypes (**C, D**) of *Ranunculusmaoxianensis* sp. nov. (**A, C**) and *R.chongzhouensis* (**B, D**), with m = median-centromeric chromosome, sm = submedian-centromeric chromosome.

During a botanical expedition to the Hengduan Mountains region from June to August 2022 for the first author’s Ph.D. dissertation project, we discovered an unusual population (Figs 1, 2) of *Ranunculus* on Jiuding Shan in Maoxian county, northwestern Sichuan province, China. The plants are somewhat similar to *R.chongzhouensis* W.T. Wang (Figs 3–10), a species also occurring in Sichuan, in having reniform leaves and puberulous receptacles, carpels and achenes, but differ by an array of characters, such as the length of hairs on the adaxial side of leaf blades, size of flowers, size and shape of petals, number of stamens, and shape of the gynoecium and aggregate fruit. Moreover, our chromosomal examination revealed that the Maoxian population in question has a chromosome number of 2*n* = 16 (Fig. 11A), while *R.chongzhouensis* has a chromosome number of 2*n* = 32 (Fig. 11B). Therefore, we determined that this population represents a hitherto undescribed species, which we describe as *R.maoxianensis* below. Furthermore, we found that *R.chongzhouensis*, the putative closest ally of *R.maoxianensis*, is much more widely distributed than documented before and that its original description is not complete, lacking a description of root and floral characters, with the description of indumentum of leaf blades, receptacles and achenes being also incorrect. Based on our critical observations of herbarium specimens and living plants in the wild, the description of this species is here emended. We also largely extended its geographical distribution.

## ﻿Materials and methods

For morphological comparison, we critically examined specimens or high-resolution specimen images of *Ranunculus* at CDBI, KUN, PE, and WCSBG (acronyms according to Thiers 2022). We also observed living plants in three populations of *R.chongzhouensis* from Sichuan and one population of the new species *R.maoxianensis* (Table 1) at flowering and fruiting time (June to July). We observed characters of roots, stems, leaves, pedicels, flowers, receptacles, petals, stamens, gynoecium, carpels, aggregate fruit and achenes, paying special attention to the indumentum of basal leaves, size of flowers, size and shape of petals, number of stamens, and the shape of gynoecium and aggregate fruit.

**Table 1.** Information about three populations of *Ranunculuschongzhou*ensis and one of *R.maoxianensis* sp. nov. observed in the wild. Populations with an asterisk were used for SEM observation of the leaf epidermis and chromosomal examination.

Taxon	Voucher	Locality
*R.chongzhouensis*	*W.Q. Fei 915* (IBSC)	China, Sichuan, Chongzhou, Jiguan Shan
	*W.Q. Fei 577* (IBSC)*	China, Sichuan, Dayi, Xiling Xue Shan
	*W.Q. Fei & H.S. Wu 395* (IBSC)	China, Sichuan, Xiaojin, Siguniang Shan
*R.maoxianensis*	W.Q. Fei 565 (IBSC)*	China, Sichuan, Maoxian, Jiuding Shan

For scanning electron microscopy (SEM), dry leaves were taken from herbarium specimens (Table 1) and mounted directly onto stubs using double-sided sellotape, gold-coated, and then observed and photographed under a JSM-6360LV scanning electron microscope.

For chromosomal examination, living plants of *Ranunculuschongzhouensis* from Xiling Xue Shan in Dayi, Sichuan, and *R.maoxianensis* from its type locality, i.e., Jiuding Shan in Maoxian, Sichuan (Table 1), were cultivated in pots in the experimental garden of South China Botanical Garden, Chinese Academy of Sciences. Root tips were pretreated in 0.1% colchicine for 2.5 h, fixed in Carnoy I (glacial acetic acid: absolute ethanol = 1: 3), then macerated in 1 M HCl at 37 °C for 45 min, and stained and squashed in Carbol fuchsin. Karyotype formulas were based on the data of measurements of mitotic-metaphase chromosomes of three cells taken from photographs. We followed the acronyms proposed by Levan et al. (1964) to describe the karyotypes.

## ﻿Results and discussion

**Figure 12.**
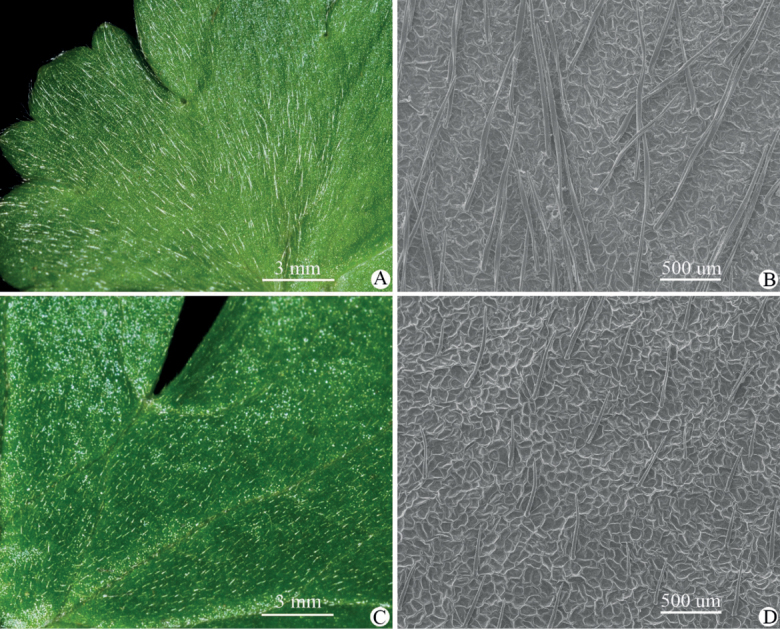
Portion of the adaxial side of the leaf blade of *Ranunculuschongzhouensis* (**A, B**) and *R.maoxianensis* sp. nov. (**C, D**), showing the difference in length of hairs. The hairs on the adaxial side of the leaf blade in *R.chongzhouensis* are 0.55‒0.85 mm long, and those in *R.maoxianensis* are 0.16‒0.28 mm long **A, C** photographed in the wild and **B, D** photographed with SEM.

Our critical observations on herbarium specimens and living plants in the wild indicate that *Ranunculusmaoxianensis* (Figs 1, 2) is morphologically similar to *R.chongzhouensis* (Figs 3–10) in having reniform leaves and puberulous receptacles, carpels and achenes, but differs by having leaves adaxially puberulous with shorter appressed hairs 0.16‒0.28 mm long (vs. longer appressed hairs 0.55‒0.85 mm long) (Fig. 12A, C), larger flowers (1.8‒2.0 cm vs. 1.4‒1.6 cm in diameter), larger (8‒10 × 5.5‒6.5 mm vs. 6‒7 × 4.5‒5 mm) and widely obovate petals (vs. obovate), more numerous stamens (35‒55 vs. 12‒18), and subglobose gynoecium and aggregate fruit (vs. ellipsoid). The difference in the indumentum of leaves between the two species is further confirmed by our SEM results (Fig. 12B, D). A detailed morphological comparison between *R.maoxianensis* and *R.chongzhouensis* is given in Table 2.

**Table 2.** Morphological comparison between *Ranunculuschongzhouensis* and *R.maoxianensis* sp. nov.

	***R.chongzhouensis***	***R.maoxianensis***
Stems	10‒25 cm tall	25‒55 cm tall
Basal leaves	5‒8, blades 2.2‒3.1 × 3.1‒3.7 cm, chartaceous, adaxially appressed puberulous with hairs 0.55‒0.85 mm long, abaxially glabrous or sometimes appressed puberulous	2‒5, blades 2.2‒3.2 × 3.8‒5.2 cm, herbaceous, adaxially appressed puberulous with hairs 0.16‒0.28 mm long, abaxially appressed puberulous
Flowers	terminal, 4‒10, 1.4‒1.6 cm in diameter	terminal, 4‒10, 1.8‒2 cm in diameter
Receptacles	3‒5 mm long, clavate, puberulous	3.5‒4 mm long, clavate, puberulous
Petals	6‒7 × 4.5‒5 mm, obovate	8‒10 × 5.5‒6.5 mm, widely obovate
Stamens	12‒18	35‒55
Gynoecium	ellipsoid	subglobose
Carpels	20‒40; ovaries ovoid or widely ovoid puberulous, styles ca. 0.9 mm long, glabrous, slightly recurved at apex	16‒22; ovaries ovoid or widely ovoid, puberulous, styles ca. 0.9 mm long, glabrous, straight or apex recurved
Aggregate fruit	ellipsoid	subglobose
Achenes	ca. 2 × 1.5 mm, obliquely or widely ovoid, puberulous, styles ca. 1 mm long, straight or apex recurved.	ca. 2.5 × 2 mm, obliquely or widely ovoid, puberulous, styles ca. 1 mm long, straight or apex recurved.

Our chromosomal examination reveals that *Ranunculusmaoxianensis* is a diploid species with 2*n* = 2*x* = 16 = 10m + 6sm (Fig. 11A, C), while *R.chongzhouensis* is a tetraploid with 2*n* = 4*x* = 32 = 16m + 16sm (Fig. 11B, D). This result lends strong support to the description of *R.maoxianensis* as a new species.

Our literature consultation and critical observations on herbarium specimens and living plants in the wild reveal that *Ranunculuschongzhouensis*, the putative closest ally of *R.maoxianensis*, lacks the description of root and floral characters, with the description of indumentum of leaf blades, receptacles and achenes by Wang (2015) being also incorrect. Wang (2015) described this species based on a single specimen, *Z.B. Feng*, *D.H. Zhu & X.J. Li 4171* (PE; Fig. 3A), from Jiguan Shan in the Anzihe Nature Reserve in Chongzhou city, Sichuan province, China. The three plants on the sheet are all fruiting, lacking roots and flowers. Therefore, the number of basal leaves and floral morphology were not mentioned in the original description of *R.chongzhouensis*. We traced two isotype sheets of *R.chongzhouensis* from WCSBG (Fig. 3B, C), which Wang (2015) did not see when he described this species as new. Wang (2015) described the basal leaves of *R.chongzhouensis* as adaxially glabrous and abaxially appressed puberulous, and the receptacles and achenes as glabrous. However, the basal leaves of this species are adaxially appressed puberulous (Fig. 3D, E) and abaxially glabrous (Fig. 3F, G), and the receptacles (Fig. 3H) and achenes (Fig. 3H, I) are puberulous. These results are further confirmed by our observations of living plants in three populations of *R.chongzhouensis*, respectively, from Chongzhou (type locality), Dayi, and Xiaojin, all in Sichuan province (Figs 4–9). In addition, we found that the indumentum on the abaxial side of the leaf blades of *R.chongzhouensis* is somewhat variable between populations. The leaf blades are often abaxially glabrous (Figs 4E, 6E), but sometimes abaxially puberulous (Fig. 8E).

From our survey of herbarium specimens and fieldwork, we found that *Ranunculuschongzhouensis* is much more widely distributed than reported by Wang (2015). In addition to its type locality, this species is also distributed in Baoxing, Dayi, Heishui, Luding, Songpan, and Xiaojin counties. Selected specimens from Baoxing (Fig. 10A), Heishui (Fig. 10B), Luding (Fig. 10C) and Songpan (Fig. 10D) are shown in Fig. 10.

*Ranunculusmaoxianensis* is readily assigned to R.sect.Ranunculus due to its swollen achenes with a distinct beak and receptacles hardly enlarged after anthesis. Wang (2015) also included *R.chongzhouensis* in this section. We accept the sectional placement of this species.

### ﻿Taxonomic treatment

#### ﻿ Ranunculus maoxianensis

Taxon classificationPlantaeRanunculalesRanunculaceae

W.Q.Fei, Q.Yuan & Q.E.Yang sp. nov.

9563309A-F228-5C26-8196-A2250FD3E7AE

urn:lsid:ipni.org:names:77313248-1

Figs 1 , 2

##### Diagnosis.

*Ranunculusmaoxianensis* is similar to *R.chongzhouensis* in leaf blade shape and indumentum of the receptacles, carpels and achenes. However, it is easily distinguishable by having leaves adaxially puberulous with shorter appressed hairs 0.16‒0.28 mm long (vs. longer appressed hairs 0.55‒0.85 mm long), larger flowers (1.8‒2 cm vs. 1.4‒1.6 cm in diameter), larger (8‒10 × 5.5‒6.5 mm vs. 6‒7 × 4.5‒5 mm) and widely obovate petals (vs. obovate), more numerous stamens (35‒55 vs. 12‒18), and subglobose gynoecium and aggregate fruit (vs. ellipsoid).

##### Type.

China. Sichuan: Maoxian, Nanxin town, Jiuding Shan, 31°30'36.28"N, 103°46'52.01"E, alt. 3274 m, in *Rhododendron* forests, 7 June 2022, *W.Q. Fei 565* (holotype: IBSC; isotypes: CDBI, IBSC, PE).

##### Description.

***Herb*** perennial, terrestrial. ***Roots*** fibrous, slender. ***Stems*** 25‒55 cm tall when in bloom, branched, erect, sparsely puberulous. ***Basal leaves*** 2‒5, long petiolate; petioles 7‒20 cm long, sparsely puberulous; blades 2.2‒3.2 × 3.8‒5.2 cm, reniform, 3-lobed or 3-partite, herbaceous, adaxially green, appressed puberulous with hairs 0.16‒0.28 mm long, abaxially light green, puberulous with hairs 0.65‒1.1 mm long, base truncate or cordate, central segment 1.2‒1.5 × 0.8‒1.2 cm, rhombic or rhombic-obovate, margin crenulate, lateral segments 1.5‒1.8 × 2‒2.6 cm, obliquely flabellate, unequally 2-partite, margin crenulate. ***Lower cauline leaves*** 1‒2, similar to basal ones but smaller. ***Upper cauline leaves*** 2‒4, subsessile or sessile, 3-sected, segments 1.5‒3 × 0.3‒0.8 mm, obtriangular, lanceolate or linear, entire or 3‒5-lobed. ***Inflorescences*** terminal, 4‒10-flowered. ***Flowers*** 1.8‒2 cm in diameter; pedicels 5‒10 cm long, appressed puberulous; receptacles 3.5‒4 mm long, clavate, puberulous; sepals 5, 4.6‒5 × 2‒3 mm, elliptic to obovate, green tinged with yellowish, adaxially glabrous, abaxially puberulous; petals 5(‒6), 8‒10 × 5.5‒6.5 mm, widely obovate, yellow, glabrous, apex rounded or subtruncate, nectary pit without a scale, claw ca. 0.6 mm long; stamens 35‒55, filaments 1.5‒2 mm long, narrowly linear, anthers 1‒1.2 mm long, oblong; gynoecium subglobose; carpels 16‒22, ovaries ca. 0.9 × 0.8 mm, ovoid or widely ovoid, laterally flattened, biconvex, puberulous, styles ca. 0.9 mm long, glabrous, straight or apex recurved. ***Aggregate fruit*** ca. 7 × 7 mm, subglobose; achenes ca. 2.5 × 2 mm, obliquely or widely ovoid, laterally flattened, biconvex, puberulous, styles ca. 1 mm long, persistent, glabrous, straight or apex recurved.

##### Etymology.

The specific epithet refers to the type locality of the new species, i.e. Maoxian county in northwestern Sichuan province, China.

##### Phenology.

Flowering from June to July; fruiting from July to August.

##### Distribution and habitat.

**Figure 13.**
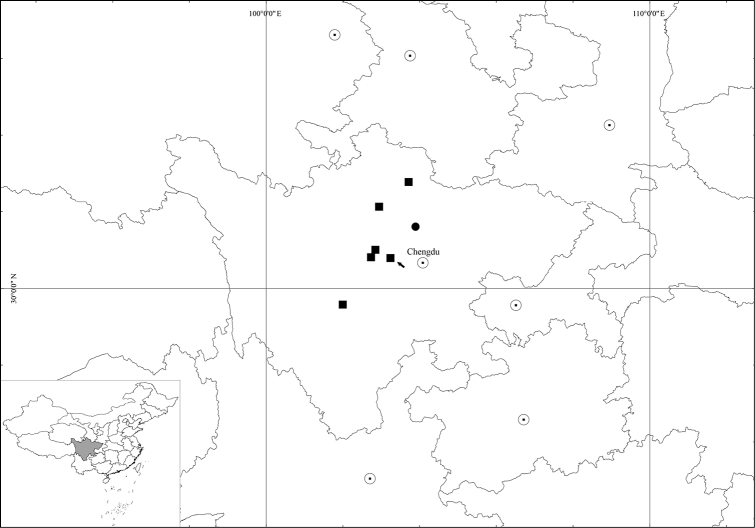
Distribution of *Ranunculuschongzhouensis* (■) and *R.maoxianensis* (●). Black arrow indicates the type locality of *R.chongzhouensis*, i.e., Chongzhou city in Sichuan province, China.

*Ranunculusmaoxianensis* is currently known only from its type locality, i.e., Jiuding Shan in Maoxian county, northwestern Sichuan province, China (Fig. 13). It grows in *Rhododendron* forests at altitudes of 3200‒3400 m above sea level.

##### Conservation status.

Although *Ranunculusmaoxianensis* is currently known only from its type locality, i.e., Jiuding Shan in Maoxian county, northwestern Sichuan province, China, our observations on living plants at its type locality revealed that this species is very common in *Rhododendron* forests at altitudes of 3200‒3400 m above sea level. It should better be categorized as “Least Concern (LC)”, following the IUCN Standards and Petitions Committee (IUCN 2022).

##### Additional specimens examined

**(paratype).** China. **Sichuan**: Maoxian, *W.Q. Fei & H.S. Wu 397* (IBSC).

#### ﻿ Ranunculus chongzhouensis

Taxon classificationPlantaeRanunculalesRanunculaceae

W.T. Wang, Bull. Bot. Res., Harbin 35(5): 645. 2015

CE2C1A7C-E94B-5A48-ADB7-735BB31B67B0

Figs 3 -10

##### Type.

China. Sichuan: Chongzhou city, Anzihe Nature Reserve, Jiguan Shan, alt. 3000 m, in forests, 27 July 2007, *Z.B. Feng*, *D.H. Zhu & X.J. Li 4171* (holotype: PE!; isotypes: WCSBG!).

##### Description.

***Herbs*** perennial, terrestrial. ***Roots*** fibrous, slender. ***Stems*** 10‒25 cm tall, ascending or erect, branched, puberulous. Basal leaves 5‒8, 3-lobed or 3-partite, long petiolate; petioles 4‒10 cm long, sparsely puberulous; blades 2.2‒3.1 × 3.1‒3.7 cm, reniform, chartaceous, adaxially green, appressed puberulous with hairs 0.55‒0.85 mm long, abaxially light green, glabrous, sometimes puberulous, base cordate, central segment 0.6‒1 × 0.9‒1.4 cm, obtrapezoid or obovate-obtrapezoid, margin 3-crenulate, lateral segments 0.8‒1.2 × 1.5‒2.3 cm, obliquely flabellate, unequally 2-lobed, margin crenulate. ***Lower cauline leaves*** 1 or absent, similar to basal ones but smaller. ***Upper cauline leaves*** 2‒3, 3-sected, subsessile, segments 1.1‒1.5 × 0.3‒0.9 mm, obliquely flabellate, lanceolate to linear, margin entire or 3‒4-lobed. ***Inflorescences*** terminal, 4‒10-flowered. ***Flowers*** 1.4‒1.6 cm in diameter; pedicels 5‒10 cm long, appressed puberulous; receptacles 3‒5 mm long, clavate, puberulous; sepals 5, 3.9‒4.5 × 2.5‒3 mm, elliptic to obovate, green tinged with yellowish, adaxially glabrous, abaxially puberulous; petals 5(‒6), 6‒7 × 4.5‒5 mm, obovate, yellow, glabrous, apex truncate or subtruncate, nectary pit without a scale, claw ca. 0.5 mm long; stamens 12‒18, filaments ca. 2 mm long, narrowly linear, anthers ca. 1 mm long, oblong; gynoecium ellipsoid; carpels 20‒40, ovaries ca. 0.9 × 0.8 mm, ovoid or widely ovoid, laterally flattened, biconvex, puberulous, styles ca. 0.9 mm long, glabrous, apex slightly recurved. ***Aggregate fruit*** ca. 7 × 5 mm, ellipsoid; achenes ca. 2 × 1.5 mm, obliquely or widely ovoid, laterally flattened, biconvex, puberulous, styles ca. 1 mm long, persistent, straight or apex recurved.

##### Phenology.

Flowering from June to July; fruiting from July to August.

##### Distribution and habitat.

*Ranunculuschongzhouensis* is distributed in Baoxing, Chongzhou, Dayi, Heishui, Luding, Songpan, and Xiaojin in Sichuan province, China (Fig. 13). It grows in forests or meadows at elevations of 2900‒4150 m above sea level.

##### Additional specimens examined.

China. **Sichuan**: Baoxing, *W.Q. Fei & H.S. Wu 371* (IBSC); Chongzhou, *W.Q. Fei 915* (IBSC), *W.B. Ju*, *L. Zhang & D.K. Chen AZH01296* (CDBI); Dayi, *W.Q. Fei 577* (IBSC), *J.P. Luo & H.M. Li 613* (IBSC), *Y.P. Zeng*, *Y.F. Luo & Y.Q. Tao 149* (IBSC); Heishui, *W.Q. Fei 719* (IBSC); Luding, *W.Q. Fei 754* (IBSC); Songpan, *W.Q. Fei 725* (IBSC); Xiaojin, *W.Q. Fei & H.S. Wu 395* (IBSC).

## ﻿Figures

## Supplementary Material

XML Treatment for Ranunculus maoxianensis

XML Treatment for Ranunculus chongzhouensis
